# Genomic landscape and mutational impacts of recurrently mutated genes in cancers

**DOI:** 10.1002/mgg3.458

**Published:** 2018-08-14

**Authors:** Baolin Liu, Fei‐Fei Hu, Qiong Zhang, Hui Hu, Zheng Ye, Qin Tang, An‐Yuan Guo

**Affiliations:** ^1^ Department of Bioinformatics and Systems Biology Key Laboratory of Molecular Biophysics of the Ministry of Education College of Life Science and Technology Huazhong University of Science and Technology Wuhan China; ^2^ Department of Biochemistry and Molecular Biology Tianjin Key Laboratory of Medical Epigenetics Tianjin Medical University Tianjin China

**Keywords:** cancer, DNA mutation, gene mutation, genomic

## Abstract

**Background:**

Cancer genes tend to be highly mutated under positive selection. Better understanding the recurrently mutated genes (RMGs) in cancer is critical for explicating the mechanisms of tumorigenesis and providing vital clues for therapy. Although some studies have investigated functional impacts of RMGs in specific cancer types, a comprehensive analysis of RMGs and their mutational impacts across cancers is still needed.

**Methods:**

We obtained data from The Cancer Genome Atlas (TCGA) and calculated mutation rate of each gene in 31 cancer types. Functional analysis was performed to identify the important signaling pathways and enriched protein types of RMGs. In order to evaluate functional impacts of RMGs, differential expression, survival, and pairwise mutation patterns analyses were performed.

**Results:**

Totally, we identified 897 RMGs and 624 of them were specifically mutant in only a single cancer type. Functional analysis demonstrated that these RMGs were enriched in hydrolases, cytoskeletal protein, and pathways like MAPK, cell cycle, PI3K‐Akt, ECM receptor interaction, and energy metabolism. The differentially expressed genes potentially affected by the same common RMG showed a relatively low overlap across different cancer types. For the 19 Mucin (MUC) family genes, nine of them were RMGs and four of them (*MUC17, MUC5B, MUC4,* and *MUC16*) were common RMGs shared in 8 to 17 cancer types. The results showed that recurrent mutations in MUC genes were significantly associated with better survival prognosis. Only a small part of RMGs was differentially expressed due to their own mutations and most of them were downregulated. In addition, pairwise mutation pattern analysis revealed the high frequency of co‐occurred mutations among RMGs in STAD.

**Conclusion:**

Through the functional analysis of RMGs, we found that six signaling pathways were disrupted in most cancer types and that energy metabolism was abnormal in tumors. The results also revealed a strong correlation between recurrently mutated genes from MUC family and human survival. In addition, gene expression and survival prognosis were associated with different mutation types of RMGs.

## 1 INTRODUCTION

DNA mutation is a driver event in cancers. The accumulation of necessary somatic mutations is a leading cause of cancer initiation and development (Vogelstein & Kinzler, 1993). Mutations in cancer genome can influence molecular function of genes and signaling pathways, leading to cell differentiation, proliferation, and survival (Hanahan & Weinberg Robert, 2011; Watson, Takahashi, Futreal, & Chin, 2013). Under positive selection, cancer genes tend to be recurrently mutated, thus showing higher mutation rates compared with background in cancers (Kandoth et al., 2013). Therefore, a deeper understanding of recurrently mutated genes (RMGs) could provide clues to better elucidate biological mechanisms of tumorigenesis and identify biomarkers for diagnosis and therapy.

Large‐scale cancer genomics projects like International Cancer Genome Consortium (ICGC) and The Cancer Genome Atlas (TCGA) provide opportunities for the integrative analysis in pan‐cancer at multiple omics levels (Gong et al., 2017). *TP53* (OMIM *191170) was reported as the most frequently mutated gene in diverse cancers, and patients with *TP53* mutation tend to have worse prognosis (Wang & Sun, 2017). Kandoth et al. investigated 127 significantly mutated genes in 12 cancers and categorized them into 20 cellular processes, including Wnt/β‐catenin, MAPK, and PI3K signaling pathways (Kandoth et al., 2013). TCGA Research Network also explored the RMGs in multiple cancers. For instance, 10 RMGs including *KRAS* (* 190070)*, TP53, CDKN2A* (* 600160)*,* and *RREB1* (* 602209) were identified in Pancreatic Ductal Adenocarcinoma (PDAC), and it was revealed that the frequent disruptions in RAS‐MAPK pathway played a pivotal role in this cancer (Network, 2014). Besides, dozens of significantly mutated genes in various canonical signaling pathways were identified in Muscle‐Invasive Bladder Cancer (BLCA), which highlighted the importance of these pathways in the disease (Robertson et al., 2017). Collectively, these findings reveal diverse functions of RMGs in cancers. However, most of these studies analyzed RMGs in a single cancer or investigated a specific RMG in cancers, so the analysis of RMGs on pan‐cancer level should be conducted to explore their common and unique features.

Several studies have investigated the impacts of recurrent mutations on gene expression and prognosis. A method named TieDIE was developed to evaluate the connection between mutations and transcriptional states and identify key signaling pathways as well as interlinking genes (Paull et al., 2013). According to the assessment of somatic coding mutations, it was realized that amino acid‐altering and truncation mutations were the most important factor that affected gene expression (Jia & Zhao, 2017). Besides, it was reported that the mutations of six RMGs including *TP53, KDR* (* 191306)*, PIK3CA* (* 171834)*, ATM* (* 607585)*, AKT1* (* 164730), and *KIT* (* 164920) were associated with a poor prognosis in sporadic triple negative breast cancer (Pop et al., 2018). The diagnostic and prognostic impacts of RMGs (e.g., *EZH2* (* 601573)*, ELP3* (* 612722), and *IDH2* (* 147650)) in lymphoma were surveyed for better clinical decision making (Rosenquist et al., 2016). Moreover, RMGs (e.g., *TET2* (* 612839)*, DNMT3A* (* 602769)*, BAP1* (* 603089), and *ASXL1* (* 612990)) involved in histone modification, chromatin remodeling and DNA methylation were associated with adverse outcome in thymic carcinoma (Wang et al., 2014). Although some studies have identified the RMGs and investigated their roles in a specific cancer type, a systematic analysis of RMGs and the mutation impacts on gene expression and prognosis across cancers is still needed.

In this work, to survey and depict a comprehensive landscape of RMGs, firstly we identified 897 RMGs spanning 31 cancer types, and investigated their functional types, distribution of mutation rates as well as signaling pathways. Then we analyzed the common RMGs (cRMGs) and MUC family genes that were significantly enriched in the RMGs. In addition, we also assessed the impacts of different mutation types on gene expression and prognosis. Finally, we chose STAD as an example to check and analyze the pairwise mutation patterns. In general, this work systematically investigated RMGs and their functions through pan‐cancer analysis, which provided clues to reveal the mechanisms of carcinogenesis and identify therapy targets.

## 2 MATERIALS AND METHODS

### 2.1 Materials

In this study, we downloaded MAF (mutation annotation file) data, mRNA expression data and survival data for 31 cancer types from FireBrowse (Center BITGDA, 2016). These cancers include adrenocortical carcinoma (ACC), bladder urothelial carcinoma (BLCA), breast invasive carcinoma (BRCA), cervical and endocervical cancers (CESC), cholangiocarcinoma (CHOL), lymphoid neoplasm diffuse large B‐cell lymphoma (DLBC), esophageal carcinoma (ESCA), glioblastoma multiforme (GBM), glioma (GBMLGG), head and neck squamous cell carcinoma (HNSC), kidney chromophobe (KICH), pan‐kidney cohort (KIPAN), kidney renal clear cell carcinoma (KIRC), kidney renal papillary cell carcinoma (KIRP), acute myeloid leukemia (LAML), brain lower grade glioma (LGG), liver hepatocellular carcinoma (LIHC), lung adenocarcinoma (LUAD), lung squamous cell carcinoma (LUSC), ovarian serous cystadenocarcinoma (OV), pancreatic adenocarcinoma (PAAD), prostate adenocarcinoma (PRAD), sarcoma (SARC), skin cutaneous melanoma (SKCM), stomach adenocarcinoma (STAD), stomach and esophageal carcinoma (STES), testicular germ cell tumors (TGCT), thyroid carcinoma (THCA), thymoma (THYM), uterine carcinosarcoma (UCS), and uveal melanoma (UVM).

### 2.2 Identification of RMGs and mutation analysis

We calculated the mutation ratio (number of mutated samples/number of total samples) of genes in each cancer type, excluding silent mutations that did not change amino acid sequences. To identify the RMGs, the threshold of mutation ratio was set as 10%. If a RMG existed in at least one‐quarter (*n* = 8) cancer types, we defined it as common RMG (cRMG).

All the mutations were categorized into MS (including missense and in‐frame mutations) and NS (including nonsense, frame‐shift and splice site mutations) types for RMGs with at least five samples in MS or NS mutation type. To survey the pairwise mutation patterns among RMGs in STAD, we used the R package “maftools” to examine the mutual exclusivity and co‐occurrence (Mayakonda & Koeffler, 2016). Mutually exclusive gene sets were identified using the function somatic interactions.

### 2.3 Functional category for RMGs

We classified the sets of RMGs into different protein categories by PANTHER Classification system (Mi, Muruganujan, Casagrande, & Thomas, 2013). The significance of each protein type in cancers was tested by Chi‐square test with *p* < 0.05. The lists of oncogenes and tumor suppressor genes were downloaded from the oncogene database (Wishart et al., 2017) and TSGene database (Zhao, Kim, Mitra, Zhao, & Zhao, 2016). To assess the functional effects of gene sets, we performed Gene Ontology (GO) and KEGG pathway enrichment analysis via DAVID (https://david.ncifcrf.gov/) (Huang, Sherman, & Lempicki, 2008, 2009).

### 2.4 Differential expression and survival analysis

Differentially expressed genes were detected between RMG‐mutation, RMG‐MS or RMG‐NS cancers and RMG wild‐type cancers by the R package “NOISeq” (Tarazona, García‐Alcalde, Dopazo, Ferrer, & Conesa, 2011). We selected the genes with |fold‐change| >1.5 and FDR < 0.1 as significant ones.

We performed survival analysis using R package “survival” as our GSCALite web server (Liu et al., 2018). The differences of overall survival time between RMG‐mutation, RMG‐MS or RMG‐NS patients and RMG wild‐type patients were shown by KM survival curves (log‐rank test). RMGs with *p*‐value <0.05 were considered as survival correlated RMGs.

## 3 RESULTS

### 3.1 Summary and pathway analysis of RMGs in cancers

**Figure 1:**
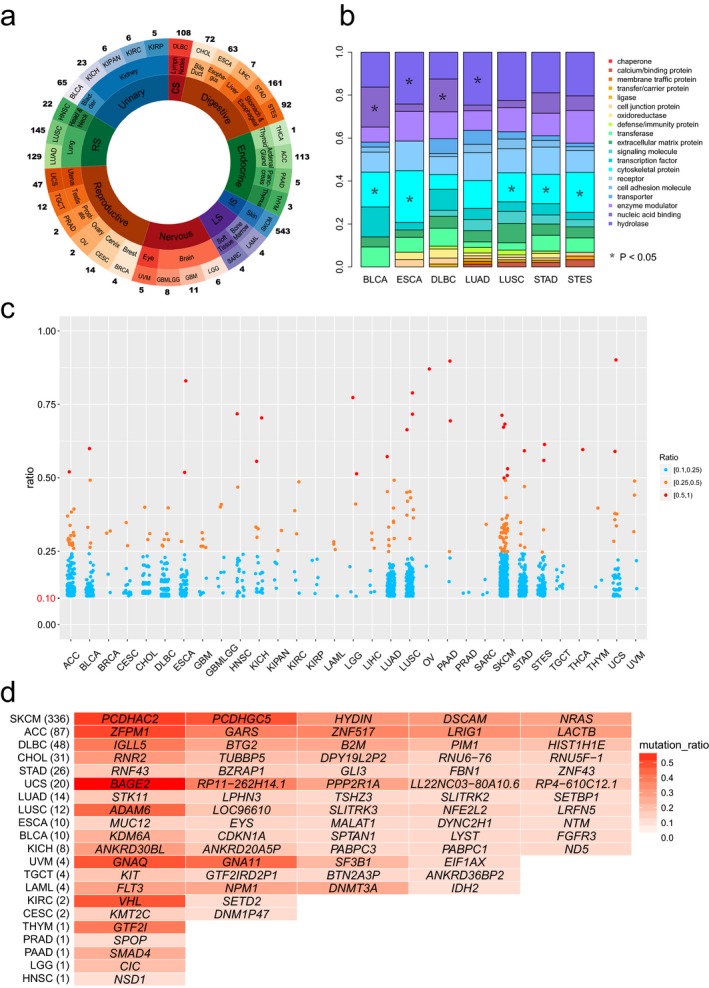
Overview of RMGs and specifically mutated RMGs in human cancer. (a) The number of RMGs in cancers. In this figure, the outermost circle means each cancer type, and the numbers outside the circle are numbers of RMGs. The middle circle represents the organ to which each cancer belong. The inner circle indicates corresponding biological system, where RS, CS, IS, and LS mean the respiratory system, circulatory system, integumentary system, and locomotor system, respectively; (b) Protein classes encoded by all RMGs. Seven cancer types with specific protein classes enriched were shown; (c) The mutation rate distribution of RMGs in cancers. Each point corresponds to one single RMG, colored in term of mutation ratios; (d) specifically mutated RMGs. The numbers in brackets mean the number of smRMGs in each cancer type. Only top five RMGs were listed for cancers with more than five smRMGs. See also Supporting Information Table S2

To survey the RMGs in cancers, we calculated mutation rates of genes in each cancer and considered genes with mutation rates >10% as RMGs. As a result, we identified 897 unique RMGs across 31 cancer types (Supporting Information Table S1). Among them, 134 genes (20.5%) were drug targets in DrugBank database (Wishart et al., 2017). The number of RMGs in each cancer type was varied from 1 to 543 (Figure 1a). There were more than 100 RMGs in SKCM, STAD, LUSC, LUAD, ACC, and DLBC (see the abbreviations of cancer types in method section), indicating that these cancers were closely related to gene recurrent mutations. However, there were less than three RMGs in cancers THYM, OV, PRAD, and THCA. The numbers of RMGs in two lung cancers (LUAD and LUSC) were similar, whereas they were very different in STAD and ESCA, two cancers from digestive system.

We classified the 897 RMGs into 20 categories according to their protein functions. Of which, 10.14% encoded hydrolases, 9.4% encoded nucleic‐acid binding proteins, and the products of the other RMGs were enzyme modulators, transporters, cell adhesion molecules, receptors, cytoskeletal proteins, etc. (Supporting Information Figure S1). The compositions of these protein categories encoded by RMGs vary among cancers (Figure 1b). Intriguingly, the RMGs in BLCA, ESCA, LUSC, STAD, and STES were significantly enriched (*p* < 0.05, Chi‐square test) in cytoskeletal protein, suggesting the tumor cell morphology has undergone major changes in these cancer types. Results showed that RMGs in BLCA and DLBC were enriched in nucleic acid binding, whereas in ESCA and LUAD, they were enriched in hydrolase. Gene Ontology functional enrichment analysis also identified 121 RMGs that were enriched in ATP binding and ATPase activity (*p*‐value < 10^−8^), and this revealed the abnormal energy metabolism in cancer.

The distribution of mutation rates of RMGs across cancers is presented in Figure 1c. While mutation rates of most RMGs (90.59%) were ranged from 10%–25%, *TP53* showed extremely high mutation rates in multiple cancer types. Specially, its mutation rate was incredibly more than 75% in UCS, OV, ESCA, and LUSC. Except for *TP53*, there were only two genes with mutation rates greater than 75%, *IPH1* (77%) in brain lower grade glioma (LGG) and *KRAS* (90%) in pancreatic adeno‐carcinoma (PAAD). We checked the protein classes of RMGs with mutation rates greater than 25% and found there was a greater proportion of hydrolase (*p*‐value = 0.023, Chi‐square test). In addition, we identified 624 specifically mutated RMGs (smRMGs) that were only in a single cancer type (Figure 1d and Supporting Information Table S2). There were 624 unique smRMGs in 21 cancer types. SKCM possessed of the most smRMGs (*n* = 336), whereas there was only one smRMG in THYM, PRAD, LGG, and HNSC, respectively. *BAGE2* was a candidate gene that encoded tumor antigens and was the most frequently mutated smRMG, with a mutation rate of 59% in melanoma (UVM). We also detected another five smRMGs that mutated in nearly half of the samples in corresponding cancers, including *PCDHAC2* in SKCM (53%), *ZFPM1* in ACC (52%), *VHL* in KIRC (49%), *GNAQ* in UVM (49%), and *ADAM6* in LUSC (45%). These 624 smRMGs may not only play crucial roles in tumorigenesis, but also could be considered as markers for clinical diagnosis.

**Figure 2:**
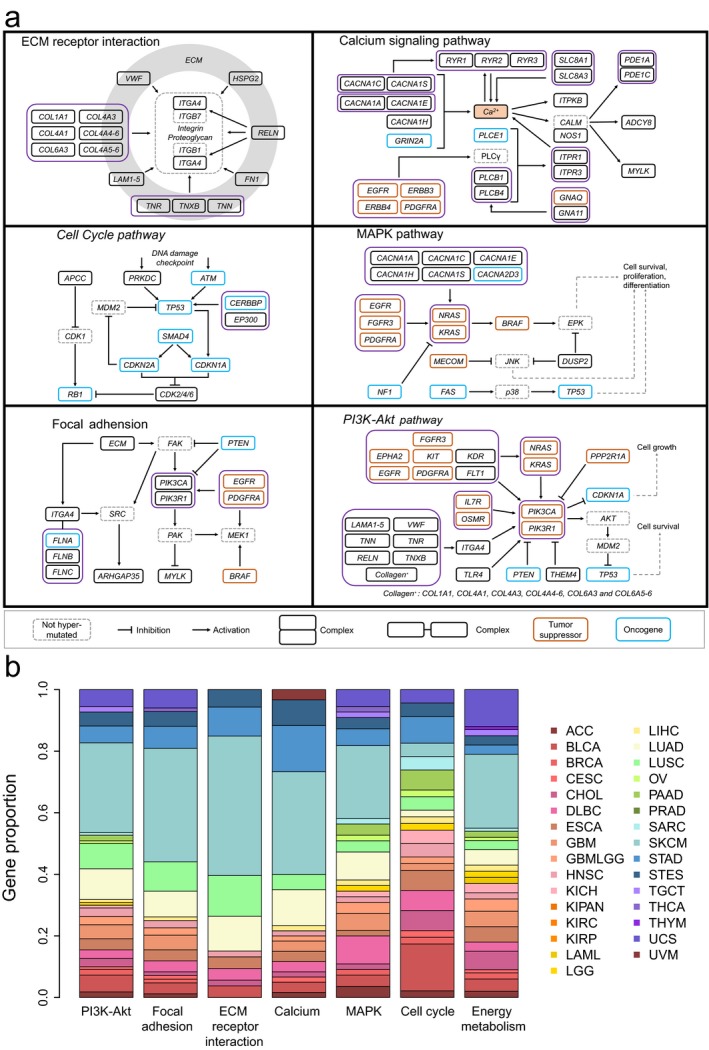
Pathway analysis of RMGs. (a) The six pathways disrupted in most cancers. A solid pane indicates a RMG, and a dashed one is not; (b) Bar plot showing the proportion of RMGs in different cancer types in each selected pathway, colored according to cancer types. The energy metabolism was additionally analyzed by merging glycolysis, TCA cycle, oxidative phosphorylation, and carbon metabolism together

Subsequently, we performed KEGG pathway analysis for all RMGs and found that six pathways were disrupted in most cancers, involving 94 RMGs (Figure 2a). PI3K‐Akt signaling pathway had the most number of RMGs (*n* = 47) and were disrupted in 23 cancer types. The disruption of cell cycle pathway indicated the abnormal process of cell division in cancer cells, and seven of the 11 RMGs this pathway were tumor suppressor genes. Strikingly, 34% genes in ECM receptor interaction pathway were recurrently mutated in cancer. SKCM harbored the most RMGs (Figure 1a) and possessed higher proportions of RMGs in these pathways than the other cancers, which was consistent with the high mutation burden in SKCM (Martincorena et al., 2015). It was also shown that BLCA had the greatest proportion of RMGs in cell cycle. We further analyzed the energy metabolism pathway by combining glycolysis, TCA cycle, oxidative phosphorylation, and carbon metabolism together. Results showed that 50 RMGs were involved in the energy metabolism pathway and that this pathway was disrupted in 25 of 31 cancer types (Figure 2b), which again support that the energy metabolism was disordered in cancer.

### 3.2 Common RMGs in cancers

**Figure 3:**
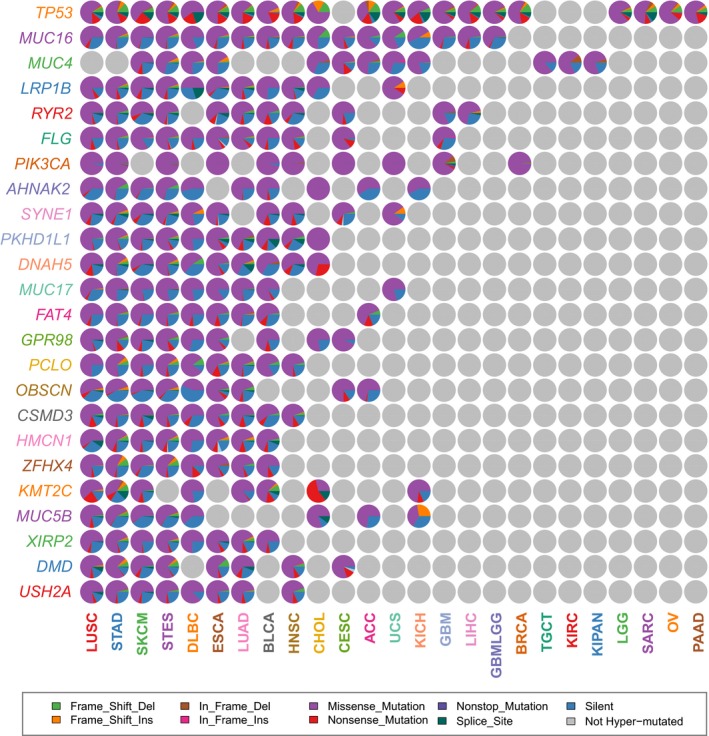
Mutation types of cRMGs in different cancer types. The pie graphs showing the proportions of mutation types in these 24 cRMGs in specific cancers, colored corresponding to mutation types

There were 24 RMGs identified in at least eight (one‐quarter) cancer types, which were considered as common RMGs (cRMGs). *TP53*,* MUC16* and *MUC4* were the top three cRMGs, recurrently mutated in 21, 17, and 12 cancer types, respectively. Figure 3a summarizes the proportions of different mutation types in each cRMG in specific cancer types. For most cRMGs, missense mutation accounts for the largest proportion (>50%), especially for *PIK3CA*. Specially, *KMT2C* in LUSC and CHOL as well as *DNAH5* in CHOL were more frequently disrupted by nonsense mutation. In addition, frame shift insertion was also found to be a key mutation for *MUC5B* in KICH, which occupied a relatively large proportion.

To analyze the functional effects of mutations, we identified differentially expressed genes (DEGs) potentially affected by each cRMG, whereas these DEGs showed a relatively low overlap across different cancer types, except for the DEGs affected by *TP53* mutations. Therefore, we made a further analysis of *TP53*. It was observed that *TP53* was more frequently disrupted by nonsense mutations, frame‐shift indels and splice site mutations compared with other cRMGs (Figure 3a), which results in the initiation and progression of cancers (Payne & Kemp, 2005; Wojnarowicz et al., 2012). Comparative analysis revealed that ratios of upregulated DEGs and downregulated DEGs affected by *TP53* could be very different in cancers (Supporting Information Figure S2). Most of DEGs were upregulated in ACC, whereas downregulation dominated these DEGs in LUSC. To assess the functional impacts of *TP53* mutations, we identified 87 DEGs shared by more than one‐quarter of the 21 cancer types. KEGG pathway analysis reveals that these gene products were significantly associated with p53 signaling pathway, cell cycle, and pathways in cancer. Some of these genes, such as *TLCD1*,* SNORD4A,* and *SLC35E3*, were the direct target genes of *TP53* as a transcription factor gene.

### 3.3 MUC family genes are enriched in RMGs and their mutations are associated with better OS prognosis

**Figure 4:**
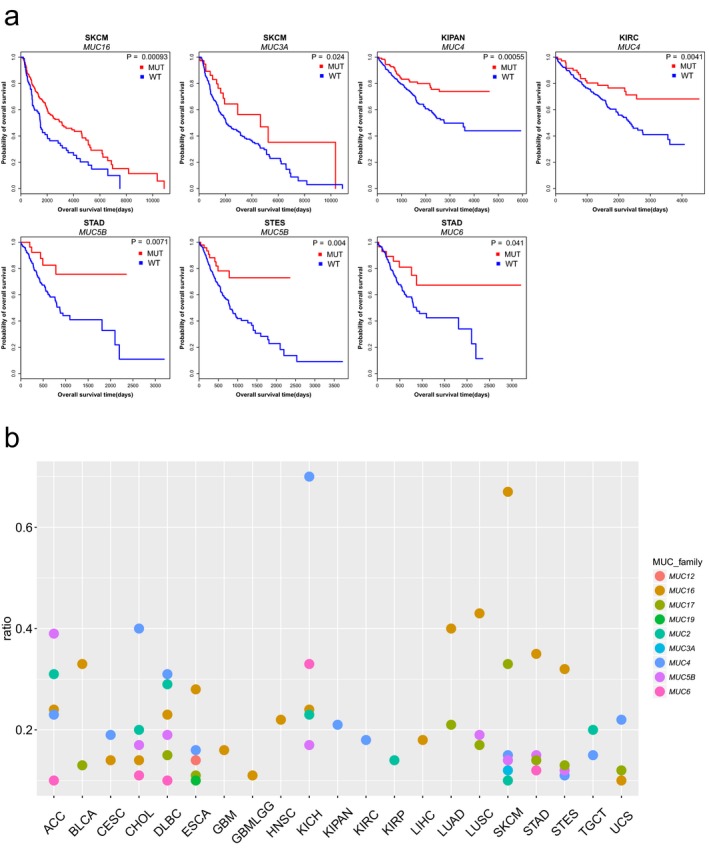
Survival prognoses and mutation rates of Mucin family genes. (a) Overall survival curve showing significant OS time between MUC‐mutated and MUC wild‐type cancers.; (b) Mutation rate distribution of 9 MUC family genes in RMGs in each cancer. Each dot corresponds to one single MUC family gene

The *MUC* family genes were significantly enriched in the RMGs (*p*‐value = 1.28 × 10^−11^, Chi‐square test), so we next analyzed this gene family. Among the 19 *MUC* family genes, nine genes are RMGs and four of them (*MUC4, MUC5B, MUC16,* and *MUC17*) were cRMGs. To explore the influence of these nine recurrently mutated *MUC* family genes on prognosis, we performed overall survival (OS) analysis based on the mutation data. Surprisingly, patients with *MUC3A*,* MUC4*,* MUC5B*,* MUC6,* and *MUC16* mutations had significantly better OS prognoses compared with those without mutations in several cancer types (Figure 4a). We then manually checked the mutation types of these five genes in corresponding cancer types. Results showed that there was a greater proportion of in‐frame deletion of *MUC4* in KIPAN and KIRC (Figure 3a), where the mutations of *MUC4* were significantly associated with prognosis. Similarly, we found a greater proportion of frame‐shift deletion of *MUC5B* in STAD and STES.

We further examined the mutation rates of recurrently mutated MUC family genes in diverse cancers (Figure 4b). For most MUC family genes, the mutation rates were lower than 40%, whereas the mutation rates of *MUC4* (in KICH) and *MUC16* (in SKCM) were greater than 60%, which could be considered as mutation signatures. Among MUC family, *MUC16* and *MUC4* were the top two RMGs identified from most cancer types (17 and 12, respectively). There were more recurrently mutated MUC family genes in SKCM, DLBC, CHOL, ACC, ESCA, KICH, and STAD compared with other cancer types. For each of these cancers, we combined recurrently mutated MUC genes together and performed OS analysis. As a result, the better OS prognosis were observed in SKCM and STAD (Supporting Information Figure S3), which further suggested that mutations of MUC family genes were frequently associated with better survival prognosis in cancers.

### 3.4 Impacts of mutation types on gene expression and prognosis

**Figure 5:**
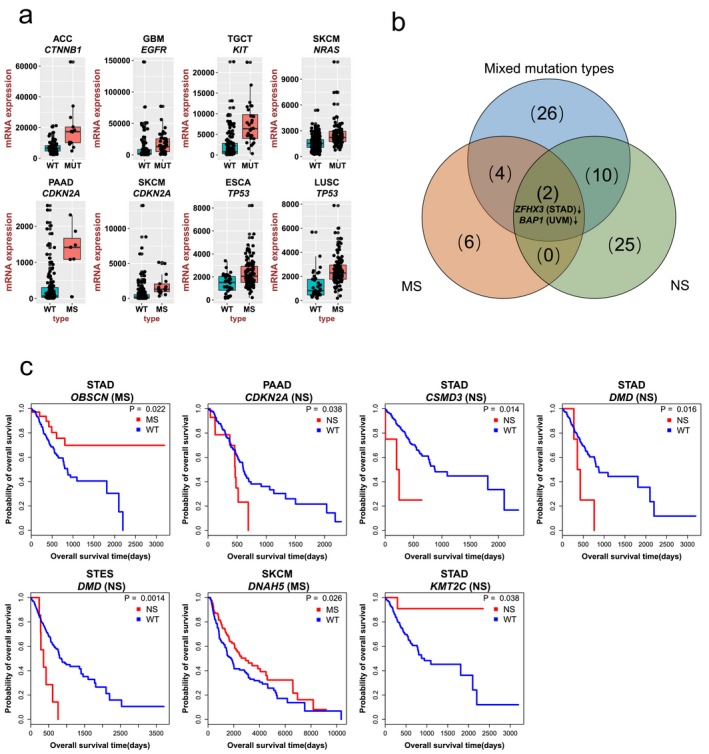
Impacts of mutation types on gene expression and prognosis. (a) Representative RMGs with significant expression change caused by their own mutations. The *x*‐axis shows the mutation type and the *y*‐axis shows mRNA expressions; (b) The Venn diagram showing differentially expressed RMGs caused by different mutation types; (c) Overall survival curves showing significant OS time differences due to specific mutation type in cRMGs

To explore the impacts of mutation types on gene expression, we firstly identified 37 RMGs which were differentially expressed caused by their own mutations (Supporting Information Table S3). There were five upregulated RMGs and 32 downregulated RMGs. Four of the five upregulated RMGs (*CTNNB1, EGFR, NRAS,* and *KIT*) were oncogenes (Figure 5a) and 12 of the 32 downregulated RMGs were tumor suppressor genes, which was consistent with the increased expression of oncogenes and decreased expression of tumor suppressor genes in tumor development.

Gene expressions were affected by different mutation types (Paull et al., 2013). We further categorized the mutations into two groups: MS (including missense and in‐frame mutations) and NS (including nonsense, frame‐shift, and splice site mutations). Subsequently, we identified 10 differentially expressed RMGs by comparing RMG‐MS cancers with RMG wild‐type cancers (Supporting Information Table S4). Two genes (*TP53* in ESCA and LUSC and *CDKN2A* in PAAD and SKCM) were upregulated (Figure 5a), whereas the other eight genes including *ABCC9, BAP1, CFH, DMD, HSPG2, TTN, ZFHX3,* and *ZFHX4* were downregulated. When comparing RMG‐NS cancers to RMG wild‐type cancers, we found all the 20 differentially expressed RMGs are downregulated (Supporting Information Figure S4), which may mainly due to nonsense‐mediated mRNA decay (Noensie & Dietz, 2001). More than half of these genes are tumor suppressor genes, including *APC*,* CDH1*,* CIC*,* FAT1*,* NF1*,* NOTCH1*,* PTEN*,* STK11*,* TP53,* and *ZFHX3*, which illustrated that tumor suppressor genes were frequently disrupted by NS. *BAP1* was reported as an epigenetic regulator, its downregulation could alter the expression of other genes, like *hTERT* whose deregulation was involved in oncogenesis (Linne et al., 2017). Venn diagram shows that *ZFHX3* gene in STAD and *BAP1* gene in UVM were consistently downregulated in these three mutation groups (Figure 5b). Both *ZFHX3* and *BAP1* are tumor suppressor genes, their decreased expression may promote the cancer development.

Similarly, to study the influences of mutation types on prognosis, we identified 13 cRMGs that mutated in sufficient samples (*n* ≥ 5) in each respective group (MS and NS) excluding *TP53* that have been widely explored (Freed‐Pastor & Prives, 2012). Furthermore, we compared OS between cRMG‐MS/cRMG‐NS patients and cRMG wild‐type patients. The results showed that six cRMGs including *OBSCN, CDKN2A, CSMD3, DMD, DNAH5,* and *KMT2C*with MS or NS mutations have significant associations with prognosis in several cancer types (Figure 5c). Patients with NS mutations in *CDKN2A* (PAAD), *CSMD3* (STAD), and *DMD* (STAD and STES) had worse survival. In particular, both *CDKN2A* and *DMD* were tumor suppressor genes, so their downregulation (Supporting Information Table S3) resulted in poor survival. *KMT2C* is a histone lysine methyltransferase. The NS mutations downregulated its mRNA expression, therefore, dysregulated transcription, chromatin architecture or cellular differentiation. Whereas, both *OBSCN* (in STAD) and *DNAH5* (in SKCM) with MS mutations had better survival. Specifically, five of the six cRMGs were not identified from our analysis by comparing OS between cRMG‐mutated patients and cRMG wild‐type patients (Supporting Information Figure S5), indicating the functional effects of RMGs were associated with specific mutation types.

### 3.5 Mutation co‐occurrence and exclusivity analysis of RMGs in STAD

**Figure 6:**
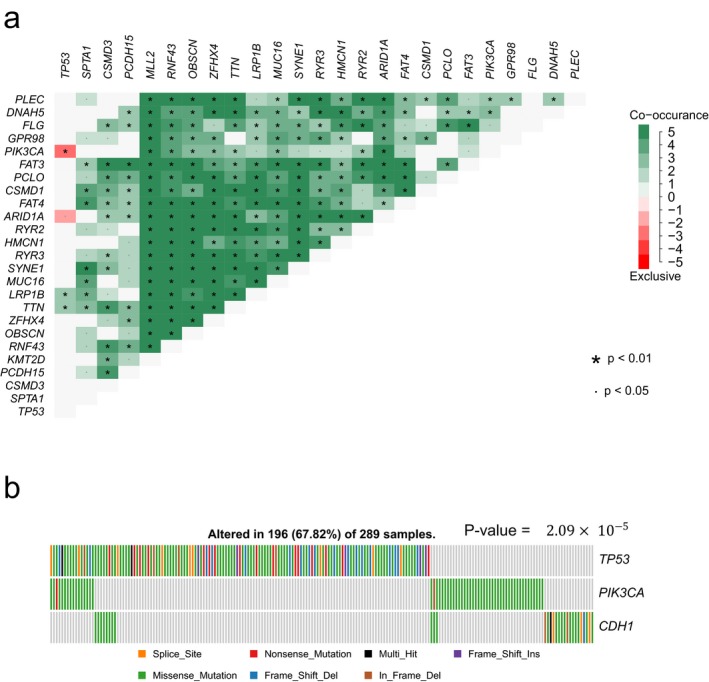
Pairwise mutation patterns among RMGs in STAD. (a) Heatmap showing mutation co‐occurrence and mutual exclusivity among top 25 mutated RMGs in STAD. Pink denotes preferential mutual exclusivity, whereas green indicates co‐mutation; (b) The gene sets with strongest mutual exclusivity. Each bar represents a sample, colored corresponding to mutation types

To have a clear understanding of pairwise mutation patterns among RMGs, we chose STAD as an example. Several studies have reported some mutually exclusive and co‐occurred gene pairs in STAD (Liang et al., 2012; Network, 2014; Zang et al., 2012). Here we focused on the pairwise mutation patterns among the top 25 RMGs (Figure 6a). Strikingly, most gene pairs among RMGs tended to be mutated together, suggesting the development of STAD requires the co‐disruption of diverse pathways. Of which, mutations in *ARID1A* and *PIK3CA* were co‐occurred (*p*‐value < 0.01), which was consistent with the previous study (Liang et al., 2012). *ARID1A* is a tumor suppressor gene belonging to the SWI/SNF family that participates in chromatin remodeling and suppresses cell proliferation (Guan et al., 2011). *PIK3CA* is a key member in PI3K pathway. Mutations in *ARID1A* could activate the PI3K pathway activity, and the concordance of mutations in *ARID1A* and PI3K pathway contributed to tumorigenesis (Liang et al., 2012). In addition, we also identified some novel co‐occurred mutations between RMG pairs. For example, *RNF43* encodes the E3 ubiquitin ligase that could inhibit Wnt signaling (Loregger et al., 2015). While the products of *FAT3* and *FAT4* are cadherins, which can bind and remove Beta‐Catenin from cytoplasmic pool for the utilization in Wnt signaling (Nelson & Nusse, 2004). The co‐occurred mutations in RNF43 and FAT3/FAT4 disrupted the Wnt signaling and thus promoted the development of cancer.

Despite the high frequency of co‐occurred mutations, 12 mutually exclusive gene sets were also identified (Table 1). Specially, *TP53, PIK3CA,* and *CDH1* are in the same or adjacent pathway, which play a pivotal role in cell proliferation, differentiation, apoptosis, and cell cycle regulation, and these three genes shows the strongest exclusivity (Figure 6b).

**Table 1:** Mutually exclusive RMG sets in STAD

Mutually exclusive gene sets	*p* value
*PIK3CA, TP53, CDH1*	2.09E‐05
*TP53, CDH1, VCAN*	1.62E‐04
*TP53, CDH1, ARID1A*	4.74E‐04
*TCHH, TP53, CDH1*	6.35E‐04
*TP53, CDH1, MYCBP2*	8.06E‐04
*PIK3CA, TP53, VCAN*	1.23E‐03
*PIK3CA, TP53, MYCBP2*	1.24E‐03
*PIK3CA, TCHH, TP53*	2.87E‐03
*TP53, VCAN, ARID1A*	3.30E‐02
*TCHH, TP53, VCAN*	4.18E‐02
*TP53, ZFHX4, CDH1*	4.25E‐02
*TP53, MYCBP2, ARID1A*	4.52E‐02

Note

*p*‐value < 0.05, fisher exact test.

## 4 DISCUSSION

In this study, we comprehensively investigated the RMGs as well as the impacts of their mutations on gene expression and prognosis across 31 cancer types. We defined the mutation rate of 10% as a threshold and identified 897 RMGs. Extensive analysis of these RMGs demonstrated multiple protein categories and signaling pathways disrupted in most cancers. Moreover, we found MUC family genes were enriched in RMGs, and mutations of five MUC family genes were associated with better OS prognosis. In addition, we assessed the impacts of mutations in RMGs on gene expression and survival, as well as the pairwise mutation patterns among RMGs.

Hydrolases including phosphatases, cathepsins, protease, glucosidase, etc., are molecular switches, which can regulate a number of signaling pathways (Stebbing et al., 2013). In this study, we showed that the RMGs with mutation rates greater than 25% were enriched in hydrolases. Among these genes, *PTEN* was a tumor suppressor gene encoding a phosphatase and its mutation may lead to decreased sensitivity to apoptosis stimulating thus promote tumorigenesis (Farrow & Mark Evers, 2003; Stambolic et al., 1998; Zhong et al., 2000). *PTPRT* encoded a member of tyrosine phosphatase, which also was reported to be common mutated (Lee et al., 2007). Mutations in *PTPRD* abrogated its function to regulate *STAT3* and promoted cancer progression (Funato, Yamazumi, Oda, & Akiyama, 2011; Zhao et al., 2010). In addition, the alterations in cathepsins may disrupt lysosomal trafficking and autophagy, thus led to tumor invasion (Dielschneider, Henson, & Gibson, 2017; White, Mehnert, & Chan, 2015). Recurrent mutations in hydrolases may cause uncontrolled proliferation, differentiation and metastasis, so target tumor cell hydrolases is a good way to treat cancer.

In this study, we analyzed the STES data, which was a combination of the STAD and ESCA data. Another merged data, glioma (GBMLGG), the combination of the GBM and LGG data, was also analyzed. STAD and ESCA (GBM and LGG) originated from the same tissue or system, so the analysis of them may show some common features and additional results (Center BITGDA, 2016). *MUC4* was reported as the major constituents of mucus, which could form gels to protect the epithelial luminal surfaces of the healthy ducts (McGuckin, Lindén, Sutton, & Florin, 2011). The recurrent mutations of *MUC4* were detected only in ESCA (16%), whereas not in STAD. However, *MUC4* was still frequently mutated in the merged data (excluding the unbalance of sample numbers), STES, which indicated this gene might play a pivotal role in both STAD and ESCA. Compared to paired normal tissues, the gene expression profile also showed the significant upregulation of *MUC4* in ESCA and STAD by using GEPIA (Tang et al., 2017). Our results suggested recurrent mutations of MUC family genes are closely associated with survival in diverse cancers, so the mutations of MUC4 may cause the increase in mRNA expression and further protect epithelial surfaces in ESCA and STAD. Similarly, *PTEN* was frequently mutated in GBM and the combined data, GBMLGG, which could also indicate the mutated *PTEN* lost its cancer suppressing property thus promote tumorigenesis in GBM.

The MS mutations may alter gene expression differently. Both *TP53* and *CDKN2A* were well‐studied tumor suppressor genes. MS mutations could alter their functions and upregulate their gene expression. Mutant p53 protein encoded by *TP53* with MS mutations could inactivate p53‐related proteins and acquire new oncogenic functions (Freed‐Pastor & Prives, 2012), so the upregulation of TP53 helped the tumor cells evade apoptosis and senescence. *CDKN2A* was a cell cycle regulatory gene that encoded *CDK4* inhibitors (Serrano, Hannon, & Beach, 1993). The mutated *CDKN2A* proteins failed to bind to *cdk4*, which promoted the development of cancer (Lilischkis, Sarcevic, Kennedy, Warlters, & Sutherland, 1996; Liu et al., 1995; Ranade et al., 1995). Besides, the MS mutations could also downregulate gene expression (Supporting Information Table S4). *DMD*,* BAP1* and *ZFHX3* were tumor suppressor genes. The decrease in their gene expression conferred a predisposition to cancer development. Whereas NS mutations could only downregulate gene expression (Supporting Information Figure S4). Using drugs or other methods to restore the expression of these potential tumor suppressor genes and epigenetic regulators, whose downregulation led to poor survival, may be a good strategy for cancer treatment. For upregulated, mutually exclusive or co‐occurred RMGs, which were associated with poor survival, might be considered as therapeutic targets (Luo, Solimini, & Elledge, 2009).

Although we analyzed the effects of mutation types on gene expression and survival, some of corresponding mechanisms were still unknown, which need more experiments to verify. For the impact of mutation types on human survival, we only focused on common RMGs, so more investigations are invited to explore this and identify novel therapeutic targets. Furthermore, the uniform pairwise mutation patterns among mutant genes in more cancer types is insufficient, which needs more efforts.

## 5 CONCLUSIONS

Overall, through function enrichment analysis of RMGs in 31 cancer types, we found six signaling pathways that disrupted in most cancer types and energy metabolism was abnormal in cancers. Strong correlation between recurrently mutated MUC family genes and human survival were revealed. In addition, we found gene expression and survival prognosis were associated with different mutation types of RMGs. These findings will help to gain a deeper understanding of tumorigenesis.

## CONFLICT OF INTEREST

The authors declare that they have no competing interests.
