# Supplementary material for: Genomic landscape and mutational impacts of recurrently mutated genes in cancers
Source: Mol Genet Genomic Med. 2018 Aug 14;6(6):910–23. doi: 10.1002/mgg3.458 (PMC6305651; doi:10.1002/mgg3.458)

Supporting information Figure S1: Protein classes of all 897 RMGs.


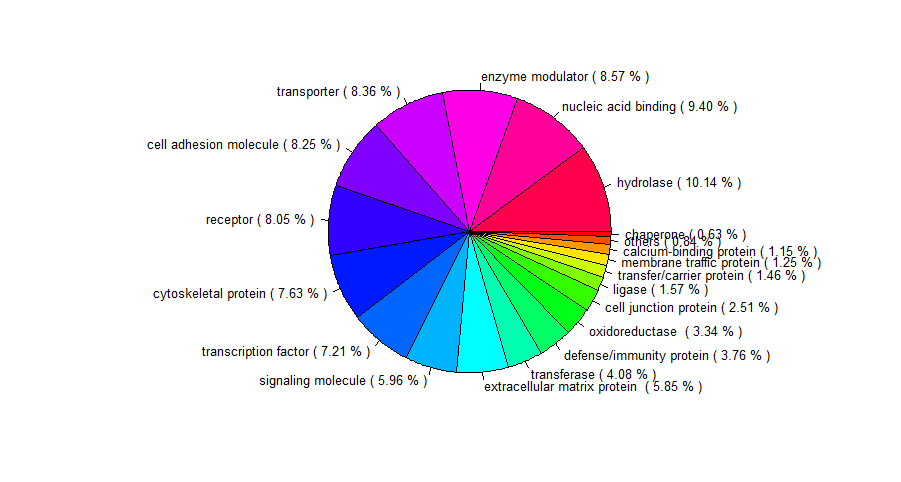


Supporting information Figure S2: Differentially expressed genes in comparisons of *TP53*-mutated patients and *TP53*-wildtype patients. The colours indicate associated logarithmic expression fold change (FDR < 0.1, |fold-change| ≥ 1.5).


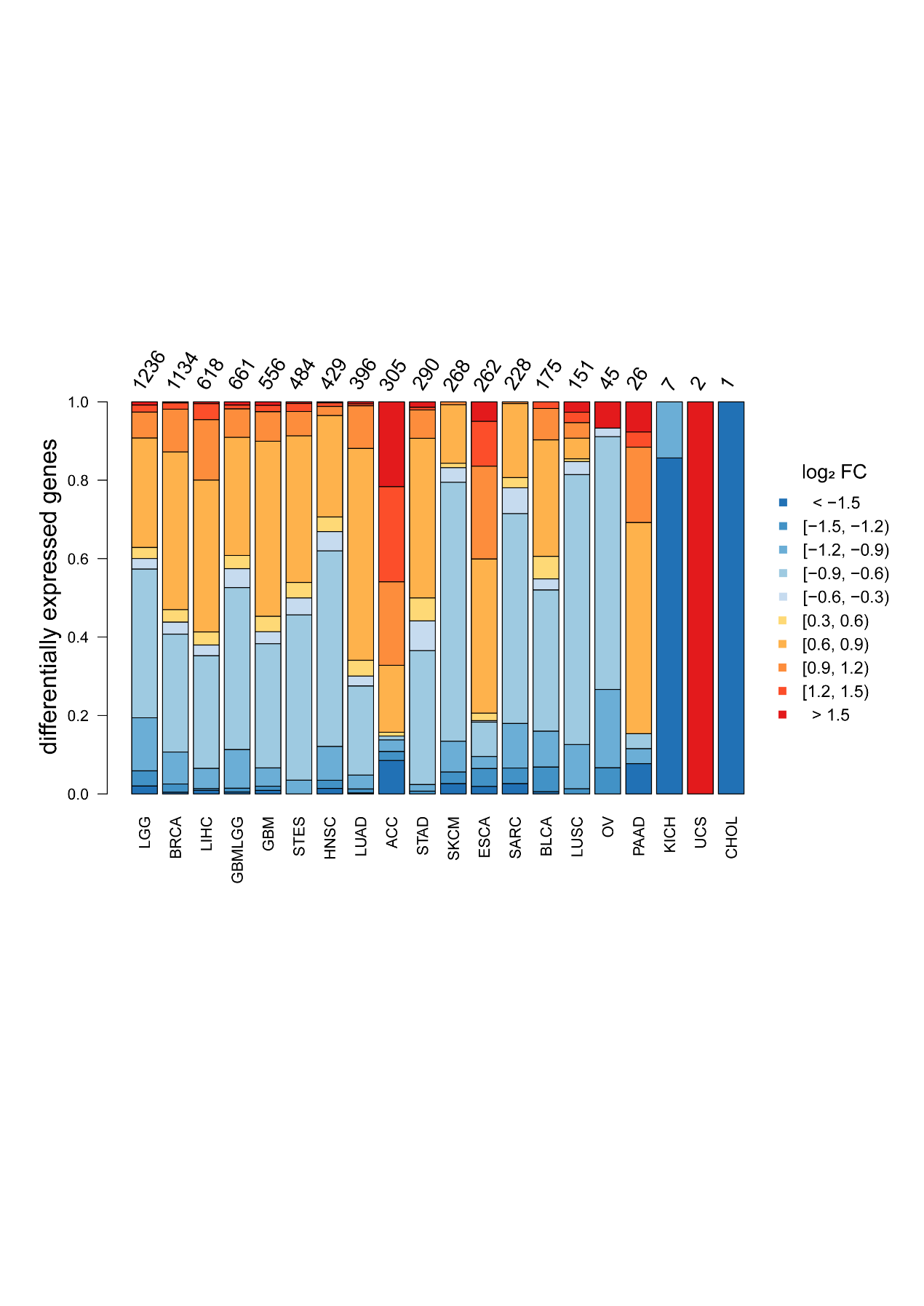


Supporting information Figure S3: Comparison of overall survival between Mucins-mutated cancers and Mucins-wildtype cancers by combining recurrently mutated Mucin family genes together (P-value < 0.05, log-rank test).


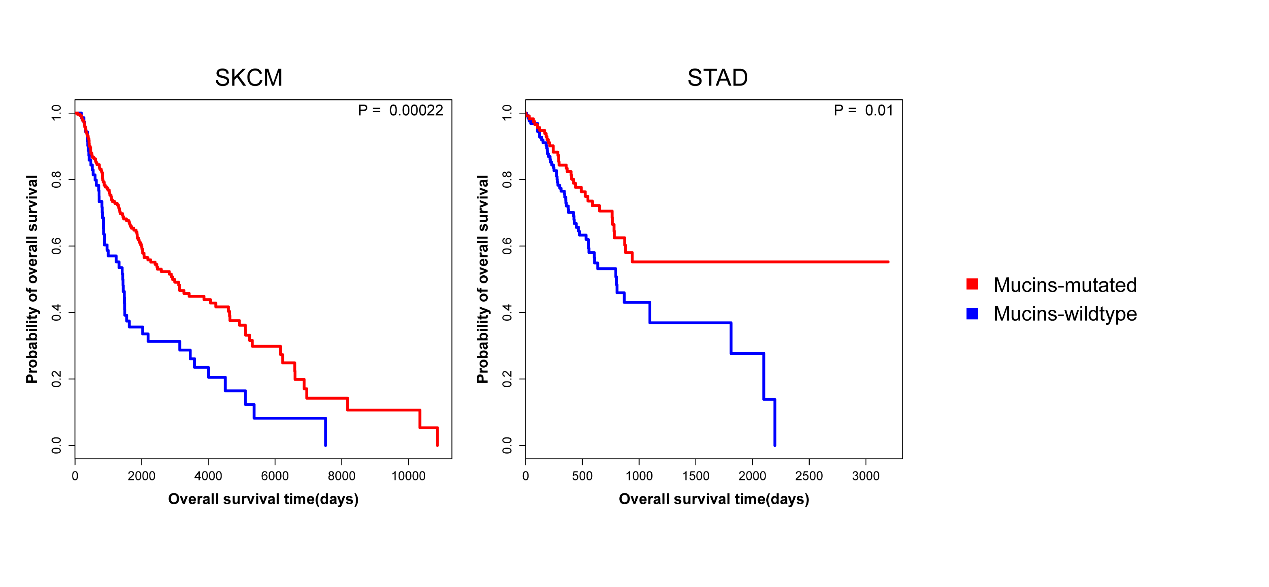


Supporting information Figure S4: The RMGs with significant expression change in comparisons of RMG-NS patients and RMG-wildtype (FDR < 0.1, |fold-change| ≥ 1.5).


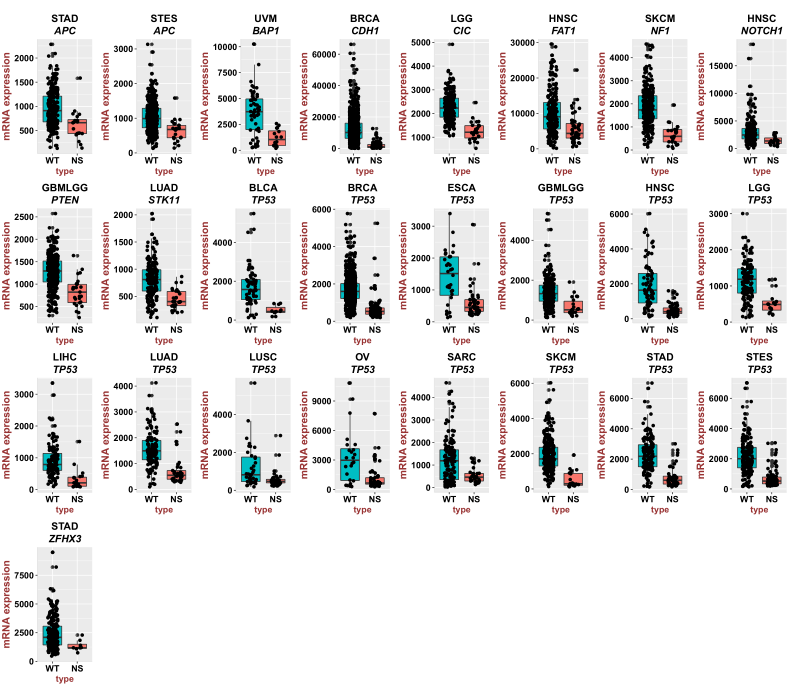


Supporting information Figure S5: Comparison of overall survival between cRMG-mutated patients and cRMG- wildtype patients (P-value < 0.05, log-rank test).


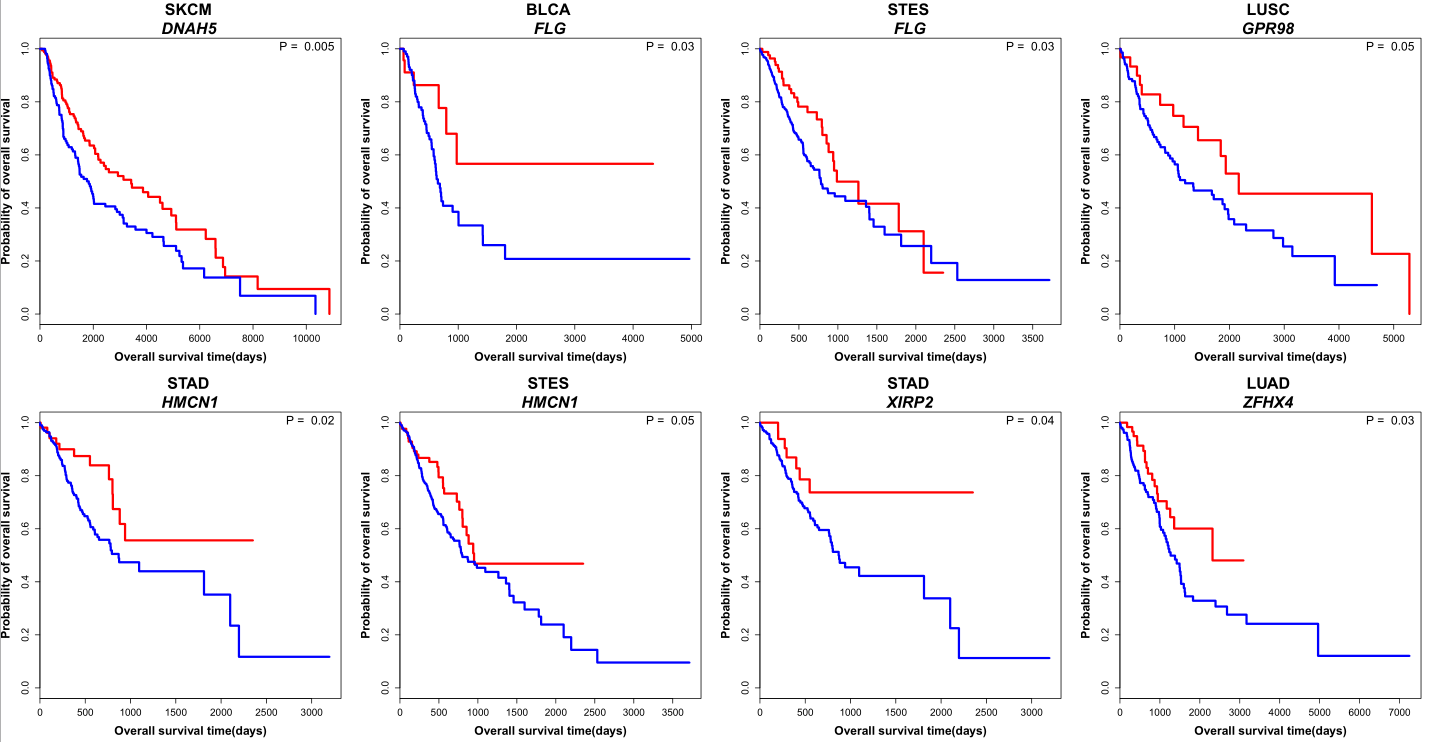

Supplement: Supplementary file 1 [file MGG3-6-910-s001.docx]
