# Supplementary material for: Genomic landscape and mutational impacts of recurrently mutated genes in cancers
Source: Mol Genet Genomic Med. 2018 Aug 14;6(6):910–23. doi: 10.1002/mgg3.458 (PMC6305651; doi:10.1002/mgg3.458)
Supplement: Supplementary file 3 [file MGG3-6-910-s003.docx]

Supporting information Table S1: The RMGs in each cancer type.

| Cancer | RMGs |
| --- | --- |
| ACC | AATK, ADAD2, ADAMTS7, AHNAK2, AMDHD1, ASB16, ASPDH, ATXN1, B3GNT6, BHLHE22, BTBD11, C16orf3, C17orf96, C19orf10, C1orf106, C2orf81, C4orf32, CACNA1A, CCDC102A, CCDC105, CCDC168, CELSR2, CLDN23, CLIC6, CRIPAK, CTNNB1, DSPP, EPPK1, ERCC2, FAM109A, FAM182A, FAM184B, FAT4, FPGS, FRG1, FRG1B, GARS, GLTPD2, GPRIN2, GPX1, HHIPL1, HLA-B, HRNR, IDUA, IRX3, KBTBD13, KCNK17, KCNN3, KNDC1, KRTAP10-6, KRTAP10-7, KRTAP4-11, KRTAP4-5, KRTAP5-5, LACTB, LRIG1, LRP11, MAL2, MAP1S, MEN1, MN1, MUC16, MUC2, MUC4, MUC5B, MUC6, NCOR2, NEFH, NMU, NOL9, NOTCH2, OBSCN, OGFR, OPRD1, OTOP1, PANK2, PKHD1, PLEC, PODXL, PROB1, RASIP1, RGMB, RGS9BP, RINL, RREB1, SALL3, SARM1, SEMA5B, SOWAHA, SP8, SPATA31C1, SYT8, TAF5, THEM4, TMEM189, TMEM247, TOR3A, TP53, TPO, TRIOBP, TSC22D2, TTN, UQCRFS1, USP42, WDR34, ZAR1, ZFPM1, ZNF517, ZNF598, ZNF628, ZNF787, ZNF814, ZZEF1 |
| BLCA | ACACA, AHNAK, AHNAK2, ANK2, ARID1A, ATM, BIRC6, CDKN1A, CELSR3, CHD7, COL7A1, CREBBP, CSMD3, DIDO1, DNAH11, DNAH3, DNAH5, EP300, ERBB3, ERCC2, FAT1, FAT3, FAT4, FBXW7, FGFR3, FLG, FLG2, FREM2, GPR98, HERC1, HMCN1, HRNR, KDM6A, LAMA2, LAMA3, LRP1B, LYST, MACF1, MLL, MLL2, MLL3, MLL4, MUC16, MUC17, NEB, PCLO, PDE4DIP, PIK3CA, PKHD1L1, RB1, RYR2, SACS, SCN1A, SPTA1, SPTAN1, SRCAP, STAG2, SYNE1, SYNE2, TP53, TTN, UBR4, VPS13D, XIRP2, ZFHX4 |
| BRCA | CDH1, PIK3CA, TP53, TTN |
| CESC | DMD, DST, ENSG00000259660, EP300, FLG, GPR98, KMT2C, KMT2D, MUC16, MUC4, OBSCN, PIK3CA, RYR2, SYNE1, SYNE2, TTN, XIST |
| CHOL | ABCA13, ADAM21P1, AHNAK, AHNAK2, AMZ2P1, ANKRD17, APC, ARID1A, ARID1B, ATM, BAGE, BAP1, BIRC6, CDC27, CLIP4, COL6A3, COX2, CYTB, DDHD1, DMXL2, DNAH5, DPY19L2P2, EP400, EPHA2, FAT1, FBN2, FMN2, FRG1, FRG1B, GLG1, GPR98, GUSBP1, HLA-B, HTT, IDH1, KIAA1109, KIF1B, LOC541471, LRP1B, LRRC37A4P, MIR548N, MLL3, MLLT10P1, MUC16, MUC2, MUC4, MUC5B, MUC6, NAV3, ND4, NLRP8, OTOP1, PAXIP1, PBRM1, PKHD1L1, PLXNA4, POLE, PRG4, PTH2, RAD54L2, RNR1, RNR2, RNU5F-1, RNU6-76, RYR3, SMG1, TCHH, TP53, TTN, TUBBP5, WDR33 |
| DLBC | ABCA7, ACACB, AHNAK2, ARHGAP21, ARID1A, ASXL3, ATM, B2M, BTG1, BTG2, CACNA1H, CARD11, CD79B, CIITA, COL21A1, CREBBP, CSMD1, CSMD3, DGKI, DMBT1, DNAH14, DNAH5, DNAH9, DNAJC13, DSG3, DSPP, DST, DUSP2, EPPK1, FAS, FAT1, FAT4, FLG, FLNC, GABRG3, GPR98, HIST1H1C, HIST1H1D, HIST1H1E, HLA-A, HLA-C, HMCN1, IGLL5, IRF4, IRF8, ITPKB, KIF1A, KLHL6, LAMA1, LAMA5, LOXHD1, LRP1B, LRP2, LTB, MLL2, MLL3, MTUS1, MUC16, MUC17, MUC2, MUC4, MUC5B, MUC6, MYD88, MYO18B, OBSCN, P2RY8, PCDH17, PCDHA10, PCDHA4, PCLO, PDE3A, PDE4DIP, PIEZO1, PIM1, PKD1, PKD1L1, PKHD1L1, POLQ, PXDNL, RHPN2, RIMS2, SALL1, SCN7A, SCN9A, SOCS1, SPEN, SPHKAP, STAT3, SYNE1, TENM4, TET2, TIAM2, TMSB4X, TNFAIP3, TP53, TTC40, TTN, UBR4, USH2A, USP17L7, VCAN, XIRP2, ZFHX4, ZNF189, ZNF208, ZNF285, ZNF814 |
| ESCA | ATM, CDKN2A, CNTNAP5, COL11A1, COL6A5, CSMD1, CSMD3, DMD, DNAH10, DNAH14, DNAH3, DNAH5, DNM1P47, DST, DYNC2H1, ENSG00000225411, ENSG00000232274, EYS, FAT3, FAT4, FLG, FMN2, GPR98, HMCN1, KMT2D, LOC101927345, LRP1B, LRRK2, MACF1, MALAT1, MT-CO1, MT-ND5, MT-RNR2, MUC12, MUC16, MUC17, MUC19, MUC4, NBEA, NEB, NTM, OBSCN, PCDH15, PCLO, PIK3CA, PKHD1L1, PTPRD, RIMS1, RIMS2, RYR2, RYR3, SNHG14, SPTA1, SSPO, SYNE1, SYNE2, TP53, TTN, UNC13C, USH2A, XIRP2, ZFHX4, ZNF804B |
| GBM | EGFR, FLG, MUC16, NF1, PIK3CA, PIK3R1, PTEN, RYR2, TP53, TTN |
| GBMLGG | ATRX, EGFR, IDH1, MUC16, PTEN, TP53, TTN |
| HNSC | CDKN2A, CSMD3, DMD, DNAH5, FAM135B, FAT1, FLG, LRP1B, MLL2, MUC16, NOTCH1, NSD1, PCLO, PIK3CA, PKHD1L1, RELN, RYR2, SI, SYNE1, TP53, TTN, USH2A |
| KICH | AHNAK2, ANKRD20A5P, ANKRD30BL, CDC27, CYTB, DDX12P, DSPP, FRG1, FRG1B, HLA-C, MIR663B, MLL3, MUC16, MUC2, MUC4, MUC5B, MUC6, NBPF10, ND5, PABPC1, PABPC3, PRSS3, TP53 |
| KIPAN | FRG1B, MUC4, PBRM1, TTN, VHL |
| KIRC | MUC4, PBRM1, SETD2, TTN, VHL |
| KIRP | FRG1B, MUC2, NEFH, TTN, TVP23C |
| LAML | DNMT3A, FLT3, IDH2, NPM1 |
| LGG | ATRX, CIC, IDH1, NOTCH1, TP53, TTN |
| LIHC | ABCA13, APOB, CTNNB1, MUC16, RYR2, TP53, TTN |
| LUAD | ABCA13, ADAMTS12, ADAMTS20, AHNAK, AHNAK2, ANK2, ANKRD30A, APOB, ASPM, ASTN1, ASXL3, BAI3, C15orf2, C1orf173, CACNA1E, CDH10, CDH12, CDH9, CNTNAP2, CNTNAP5, COL11A1, COL3A1, COL6A3, CPS1, CSMD1, CSMD2, CSMD3, CTNNA2, DMD, DNAH3, DNAH5, DNAH7, DNAH8, DNAH9, DST, EGFR, EPHA5, FAM135B, FAM47C, FAM5C, FAM75A6, FAT1, FAT3, FAT4, FBN2, FER1L6, FLG, FLG2, FLJ46321, FMN2, FRG1B, GPR112, GPR158, GRIN2A, HMCN1, HRNR, KEAP1, KRAS, LPHN3, LPPR4, LRP1B, LRP2, LRRC7, MAGEC1, MKI67, MLL3, MUC16, MUC17, MXRA5, MYH1, MYH2, MYH7, MYH8, NALCN, NAV3, NCKAP5, NEB, NF1, NLRP3, NOTCH4, NRXN1, OBSCN, PAPPA2, PCDH10, PCDH11X, PCDH15, PCLO, PEG3, PKD1L1, PKHD1L1, PLXNA4, PRDM9, PTPRD, PTPRZ1, PXDNL, RELN, RIMS2, RP1L1, RYR1, RYR2, RYR3, SETBP1, SI, SLC8A1, SLITRK2, SNTG1, SORCS1, SORCS3, SPEF2, SPTA1, STK11, SYNE2, TAF1L, TLR4, TNN, TNR, TP53, TPTE, TRPS1, TSHZ3, TTN, USH2A, XIRP2, ZFHX4, ZNF521, ZNF536, ZNF804A, ZNF831 |
| LUSC | ABCA13, ADAM6, ADAMTS12, ADAMTS20, AHNAK, AHNAK2, ALMS1, ALPK2, ANK2, ANKRD30A, APOB, ASTN2, BAI3, BIRC6, C1orf173, C20orf26, CDH10, CDH12, CDH18, CDH9, CDKN2A, CNGB3, CNTNAP2, CNTNAP5, COL11A1, COL12A1, COL22A1, COL6A6, CPS1, CRB1, CSMD1, CSMD2, CSMD3, CTNNA2, CUBN, DMD, DNAH10, DNAH11, DNAH5, DNAH7, DNAH8, DNAH9, DYNC1H1, ELTD1, FAM135B, FAM5C, FAT1, FAT3, FAT4, FBN2, FBN3, FLG, FMN2, FN1, GPR98, HCN1, HEATR7B2, HERC2, HMCN1, HRNR, KEAP1, KIAA1109, LAMA2, LCT, LOC96610, LRFN5, LRP1B, LRP2, LRRC7, LRRK2, MACF1, MAGEC1, MDN1, MLL2, MLL3, MUC16, MUC17, MUC5B, MYCBP2, MYH1, MYH2, MYH4, MYH8, NAV3, NEB, NF1, NFE2L2, NRXN1, OBSCN, ODZ1, PAPPA2, PCDH11X, PCDH15, PCLO, PDE4DIP, PEG3, PIK3CA, PKHD1, PKHD1L1, PLXNA4, PREX2, PTPRT, PXDNL, RELN, RIMS2, ROS1, RP1, RYR1, RYR2, RYR3, SCN1A, SI, SLITRK3, SORCS1, SPHKAP, SPTA1, SSPO, STAB2, SYNE1, SYNE2, TAF1L, THSD7B, TMEM132D, TNN, TNR, TP53, TPR, TPTE, TTN, UNC13C, UNC5D, USH2A, USP34, VCAN, VPS13B, WDR17, XIRP2, XIST, ZFHX4, ZNF208, ZNF536, ZNF676, ZNF804A, ZNF804B |
| OV | TP53, TTN |
| PAAD | CDKN2A, KRAS, SMAD4, TP53, TTN |
| PRAD | SPOP, TTN |
| SARC | ATRX, RB1, TP53, TTN |
| SKCM | ABCA12, ABCA13, ABCA4, ABCB1, ABCB11, ABCB5, ABCC8, ABCC9, abParts, ACAN, ACSM2B, ADAM18, ADAM28, ADAM29, ADAM7, ADAMTS12, ADAMTS18, ADAMTS19, ADAMTS20, ADAMTS6, ADAMTS9, ADCY8, ADH1B, ADH1C, AGBL1, AHNAK, AHNAK2, AKAP9, ALMS1, ALPK2, ANK1, ANK2, ANK3, ANKRD20A2, ANKRD20A9P, ANKRD30A, ANKRD30BP2, ANO4, APOB, ARAP2, ARID2, ARMC4, ARPP21, ASPM, ASTN1, ASXL3, ATP13A4, ATP13A5, ATP8B4, BAI3, BC063132, BCL11A, BCLAF1, BRAF, BSN, C10orf71, C12orf63, C15orf2, C1orf168, C1orf173, C2orf71, C3, C6, C7, C7orf58, C8A, C8B, C8orf34, CACNA1A, CACNA1C, CACNA1E, CACNA1S, CACNA2D3, CARD11, CASR, CATSPERB, CCDC141, CD163, CD163L1, CDH10, CDH18, CDH23, CDH4, CDH6, CDH9, CDKN2A, CFB, CFH, CFTR, CHD6, CHEK2P2, CHGB, CLCN1, CMYA5, CNGB3, CNTN4, CNTN5, CNTNAP2, CNTNAP4, CNTNAP5, COL11A1, COL11A2, COL12A1, COL14A1, COL19A1, COL1A1, COL21A1, COL22A1, COL28A1, COL3A1, COL4A1, COL4A3, COL4A4, COL4A5, COL4A6, COL5A1, COL5A2, COL5A3, COL6A3, COL6A6, COL7A1, CPAMD8, CR1, CRB1, CSMD1, CSMD2, CSMD3, CTNND2, CUBN, CUX2, CXorf22, CYP2C19, DAB1, DBC1, DCC, DCDC5, DCHS2, DDX60, DIDO1, DMBT1, DMD, DNAH10, DNAH11, DNAH17, DNAH2, DNAH3, DNAH5, DNAH7, DNAH8, DNAH9, DOCK3, DPYD, DSC1, DSC2, DSC3, DSCAM, DSCAML1, DSG1, DSG3, DSG4, DSP, DST, DUSP27, DYSF, EFCAB6, EGFLAM, ENAM, EPHA6, EPHA7, EPPK1, ERBB4, ERC2, EVPL, F5, F8, FAM135B, FAM47A, FAM47C, FAM5C, FAM83B, FAT2, FAT3, FAT4, FBN2, FBN3, FCGBP, FCRL5, FER1L6, FGA, FILIP1, FLG, FLG2, FLJ43315, FLNB, FLNC, FLT1, FMN2, FNDC1, FRAS1, FREM1, FREM2, FRG1B, FSIP2, GFRAL, GIMAP8, GK2, GLI2, GPC5, GPR112, GPR158, GPR179, GPR98, GPRC6A, GRIA1, GRIA2, GRID2, GRIN2A, GRIN2B, GRIN3A, GRM3, GRM7, GRM8, HCN1, HDAC9, HEATR7B2, HEATR8, HECW1, HECW2, HEPHL1, HERC2, HIVEP3, HMCN1, HRNR, HYDIN, IGSF1, IGSF10, IL7R, ITGA4, ITPR1, KALRN, KCNB1, KCNB2, KCNH5, KCNH7, KCNQ3, KCNQ5, KCNT2, KDR, KEL, KIAA1109, KIAA1210, KIAA1217, KIAA1462, KIAA2022, KIF4B, KIR2DL2, KIR3DL2, KLHDC7A, KSR2, LAMA1, LAMA2, LAMA3, LAMB4, LCT, LILRA1, LILRB4, LILRP2, LOC646214, LPA, LPHN2, LRBA, LRP1, LRP1B, LRP2, LRRC4C, LRRC7, LTBP2, MACF1, MAGEC1, MARCO, MBD5, MECOM, MED12L, MGAM, MGC70870, MLL, MLL2, MLL3, MORC1, MPP7, MST1P2, MTUS2, MUC16, MUC17, MUC2, MUC3A, MUC4, MUC5B, MUM1L1, MXRA5, MYH1, MYH13, MYH15, MYH2, MYH4, MYH7, MYH8, MYLK, MYO15A, MYO18B, MYO3A, MYO5B, MYO7B, MYOCD, MYOM2, MYOM3, MYT1L, NAV3, NBEA, NBEAL1, NBPF1, NBPF10, NCKAP5, NDST4, NEB, NEBL, NF1, NLRP11, NLRP12, NLRP13, NLRP3, NLRP4, NLRP5, NLRP8, NLRP9, NOS1, NOTCH4, NRAS, NRK, NRXN1, NRXN3, NUP210L, NWD1, NYAP2, OBSCN, ODZ1, ODZ2, ODZ3, ODZ4, OGDHL, OR4K1, OR4M1, OR51S1, OSMR, OTOF, OTOGL, PAK7, PAPPA, PAPPA2, PCDH15, PCDH18, PCDHAC2, PCDHB3, PCDHB4, PCDHB5, PCDHB7, PCDHB8, PCDHGC5, PCLO, PCNXL2, PCSK5, PDE1A, PDE1C, PDE4DIP, PDGFRA, PDZD2, PEG3, PGK2, PHLDB2, PIK3C2G, PKD1L1, PKHD1, PKHD1L1, PLCB1, PLCB4, PLCE1, PLCH1, PLXNA4, POM121L12, POTEG, PPFIA2, PPP1R3A, PRB2, PRDM9, PREX2, PRUNE2, PSG3, PSG4, PTCHD2, PTPRB, PTPRC, PTPRD, PTPRK, PTPRN2, PTPRT, PXDNL, RACGAP1P, RBP3, RELN, RFX6, RGPD3, RGPD4, RGS7, RIMBP2, RIMS1, RIMS2, RNF17, ROBO2, ROS1, RP1, RP1L1, RPTN, RUNX1T1, RYR1, RYR2, RYR3, SACS, SALL1, SAMD9, SAMD9L, SCAND3, SCN10A, SCN11A, SCN1A, SCN2A, SCN3A, SCN5A, SCN7A, SCN9A, SDK1, SELP, SERPINB3, SEZ6L, SH3RF2, SI, SIPA1L1, SLC15A2, SLC38A4, SLC8A3, SLC9A4, SLC9C1, SLC9C2, SLCO1B3, SLCO6A1, SLIT2, SLIT3, SNCAIP, SORCS3, SORL1, SPAG17, SPEF2, SPEG, SPEN, SPHKAP, SPTA1, SPTB, SRGAP3, SSPO, ST18, ST6GAL2, STAB1, STAB2, STK31, STON1-GTF2A1L, STXBP5L, SVEP1, SYNE1, SYNPO2, TACC2, TAF1L, TCEB3B, TCHHL1, TCRA, TDRD5, TECTA, TEX15, TG, THEMIS, THSD7B, TLL1, TLR4, TMC5, TMEM132B, TMEM132D, TNN, TNR, TNXB, TP53, TP63, TPO, TPTE, TPTE2, TRANK1, TRHDE, TRIOBP, TRPC4, TRPM6, TRRAP, TSHZ2, TSIX, TTN, UBR4, UGT1A1, UGT2A1, UGT2B10, UGT2B4, UNC13C, UNC79, USH2A, USP29, VCAN, VPS13B, VPS13D, VWF, WBSCR17, X97876, XDH, XIRP1, XIRP2, XIST, ZAN, ZBBX, ZDBF2, ZFHX3, ZFHX4, ZFPM2, ZNF208, ZNF536, ZNF560, ZNF676, ZNF804A, ZNF831, ZNF99 |
| STAD | ABCA13, AHNAK2, AKAP9, ANK2, ANK3, APC, APOB, ARID1A, ASH1L, ASPM, ASTN2, ATM, BAI3, BZRAP1, C12orf51, CACNA1C, CACNA1E, CDH1, CDH23, CELSR1, CELSR3, CHD6, CMYA5, CNTNAP2, COL11A1, COL12A1, CREBBP, CSMD1, CSMD2, CSMD3, CTNND2, CUBN, DCC, DCHS1, DCHS2, DCLK1, DIDO1, DMD, DNAH10, DNAH11, DNAH2, DNAH3, DNAH5, DNAH7, DNAH8, DNAH9, DST, EP400, ERBB3, ERBB4, FAT1, FAT2, FAT3, FAT4, FBN1, FLG, FMN2, FREM2, GLI3, GPR98, HERC2, HMCN1, HRNR, HSPG2, IGSF10, ITPR3, KALRN, KIAA1109, LAMA1, LRP1, LRP1B, LRP2, LRRK2, MACF1, MAP2, MDN1, MED12L, MLL, MLL2, MLL3, MUC16, MUC17, MUC5B, MUC6, MYCBP2, MYH10, MYLK, NALCN, NAV3, NBEA, NEB, NRXN1, NRXN3, OBSCN, ODZ1, ODZ3, PCDH10, PCDH15, PCDH17, PCLO, PDE4DIP, PDZD2, PEG3, PIK3CA, PKHD1L1, PLEC, PLXNA4, POLQ, PREX2, PRKDC, PSME4, PTPRD, PTPRT, PXDN, PXDNL, RELN, REV3L, RIMS2, RNF213, RNF43, ROBO2, RP1, RYR1, RYR2, RYR3, SACS, SCN3A, SDK1, SLITRK5, SPEN, SPTA1, SRCAP, SSPO, SYNE1, SYNE2, TACC2, TCHH, TECTA, TG, TNXB, TP53, TRIO, TRPS1, TRRAP, TTN, UNC13C, USH2A, USP34, UTRN, VCAN, VPS13A, VPS13B, WDFY3, XIRP2, XYLT2, ZC3H13, ZFHX3, ZFHX4, ZNF43, ZNF536, ZNF804B |
| STES | ABCA13, AHNAK2, ANK2, ANK3, APC, APOB, ARID1A, ATM, BZRAP1, CACNA1E, CMYA5, COL11A1, COL12A1, CSMD1, CSMD2, CSMD3, CUBN, DCHS2, DMD, DNAH10, DNAH11, DNAH3, DNAH5, DNAH7, DNAH8, DNAH9, DST, DYNC2H1, ERBB4, FAT2, FAT3, FAT4, FBN1, FLG, FMN2, GLI3, GPR98, HERC2, HMCN1, KIAA1109, LAMA1, LRP1B, LRP2, LRRK2, MACF1, MDN1, MED12L, MLL2, MUC16, MUC17, MUC4, MUC5B, MYCBP2, NALCN, NAV3, NBEA, NEB, NRXN1, OBSCN, PCDH10, PCDH15, PCLO, PDZD2, PIK3CA, PKHD1L1, PLEC, PLXNA4, PREX2, PTPRD, RELN, RIMS1, RIMS2, RNF43, RP1, RYR1, RYR2, RYR3, SACS, SDK1, SPTA1, SSPO, SYNE1, SYNE2, TG, TP53, TRRAP, TTN, UNC13C, USH2A, XIRP2, ZFHX4, ZNF804B |
| TGCT | ANKRD36BP2, BAGE, BTN2A3P, FRG1B, GTF2IRD2P1, KIT, KRAS, MUC2, MUC4, SEC22B, TVP23C |
| THCA | BRAF |
| THYM | DNM1P47, GTF2I, MT-RNR2 |
| UCS | AHNAK, ANKRD20A8P, ARHGAP35, ARID1A, BAGE2, CHD4, CROCCP2, ESPNP, FBXW7, FLNA, FRG1B, KMT2D, KRAS, LL22NC03-80A10.6, LRP1B, MGAM, MT-CO1, MT-CO3, MT-CYB, MT-ND1, MT-ND4, MT-ND5, MT-RNR2, MUC16, MUC17, MUC4, NBPF10, PIK3CA, PIK3R1, PPP2R1A, PTEN, RB1, RP11-262H14.1, RP11-417J8.6, RP11-423O2.5, RP11-764K9.1, RP4-610C12.1, SEC22B, SNHG14, SYNE1, TP53, TPTE2P6, TTN, VPS13B, ZAN, ZBTB7B, ZNF814 |
| UVM | BAP1, EIF1AX, GNA11, GNAQ, SF3B1 |

Supporting information Table S3: The RMGs with significant expression change in comparisons of RMG-mutated cancers and RMG-wildtype cancers (FDR < 0.1, |fold-change| ≥ 1.5).

| Genes | Cancers | Diff | log2FC |
| --- | --- | --- | --- |
| CTNNB1 | ACC | UP | 1.43 |
| CDH1 | BRCA | DOWN | -2.17 |
| TET2 | DLBC | DOWN | -1.17 |
| RYR3 | ESCA | DOWN | -2.3 |
| EGFR | GBM | UP | 0.87 |
| ATRX | GBMLGG | DOWN | -0.72 |
| IDH1 | GBMLGG | DOWN | -0.69 |
| FAT1 | HNSC | DOWN | -0.83 |
| NEFH | KIRP | DOWN | -2.55 |
| ATRX | LGG | DOWN | -1.08 |
| CIC | LGG | DOWN | -0.66 |
| CDKN2A | PAAD | UP | 1.39 |
| ATRX | SARC | DOWN | -0.84 |
| CFH | SKCM | DOWN | -1 |
| NF1 | SKCM | DOWN | -0.79 |
| NRAS | SKCM | UP | 0.73 |
| TP53 | SKCM | DOWN | -0.81 |
| ATM | STAD | DOWN | -0.59 |
| CDH1 | STAD | DOWN | -0.66 |
| CELSR3 | STAD | DOWN | -0.66 |
| DCHS1 | STAD | DOWN | -0.81 |
| DST | STAD | DOWN | -0.74 |
| FAT2 | STAD | DOWN | -3.17 |
| FBN1 | STAD | DOWN | -0.59 |
| HSPG2 | STAD | DOWN | -0.84 |
| KALRN | STAD | DOWN | -0.71 |
| LRP1 | STAD | DOWN | -0.91 |
| MAP2 | STAD | DOWN | -1 |
| MYH10 | STAD | DOWN | -0.69 |
| PCDH17 | STAD | DOWN | -0.76 |
| RNF43 | STAD | DOWN | -0.75 |
| SCN3A | STAD | DOWN | -1.39 |
| SDK1 | STAD | DOWN | -0.82 |
| XYLT2 | STAD | DOWN | -0.59 |
| ZC3H13 | STAD | DOWN | -0.6 |
| ZFHX3 | STAD | DOWN | -0.72 |
| ZFHX4 | STAD | DOWN | -0.86 |
| ZNF43 | STAD | DOWN | -1.1 |
| RNF43 | STES | DOWN | -0.66 |
| ZFHX4 | STES | DOWN | -0.69 |
| KIT | TGCT | UP | 1.75 |
| BAP1 | UVM | DOWN | -1.46 |

Supporting information Table S4: The RMGs with significant expression change in comparisons of RMG-MS cancers and RMG-wildtype cancers (FDR < 0.1, |fold-change| ≥ 1.5).

| Genes | Cancers | Diff | log2FC |
| --- | --- | --- | --- |
| TP53 | ESCA | UP | 0.75 |
| TP53 | LUSC | UP | 1.04 |
| CDKN2A | PAAD | UP | 2.14 |
| TTN | PRAD | DOWN | -2.49 |
| ABCC9 | SKCM | DOWN | -0.99 |
| CDKN2A | SKCM | UP | 1.74 |
| CFH | SKCM | DOWN | -1.03 |
| DMD | STAD | DOWN | -1.13 |
| HSPG2 | STAD | DOWN | -0.93 |
| ZFHX3 | STAD | DOWN | -0.61 |
| ZFHX4 | STAD | DOWN | -0.81 |
| BAP1 | UVM | DOWN | -0.98 |
